# Middlesex Hospital

**Published:** 1852-11

**Authors:** 


Middlesex Hospital. *Physicians*—Drs. F. Hawkins, Crawford, and S. Thompson. *Physician Accoucheur*—Dr. Frere. *Assistant Physician*—Dr. Stewart. *Surgeons*—Messrs. Shaw, De Morgan, and Moore. *Assistant Surgeon*—Mr. Henry. *Surgeon Dentist*—Mr. Tomes. *Apothecary*—Mr. Corfe.

Medical and Surgical School. *Winter Session.*—Physiology and General Anatomy, Mr. De Morgan: Anatomy, Descriptive and Surgical, Mr. Moore. Practical Anatomy, Mr. Nunn and Dr. Van der Byl. Chemistry, Mr. T. Taylor, and Heisch. Medicine, Drs. Crawford and S. Thompson. Surgery, Mr. Shaw. Morbid Anatomy, Mr. Henry. Practical Pharmacy, with Dispensing, Mr. Corfe. *Summer Session.*—Materia Medica, &c., Dr. Stewart. Midwifery, Dr. Frere. Botany, Mr. Bentley. Medical Jurisprudence, Dr. Goodfellow. Demonstrations in Anatomy, Mr. Nunn. Comparative Anatomy, Mr. Waterhouse. Practical Chemistry, Messrs. Taylor and Heisch.

